# Coagulation Traits of Sheep and Goat Milk

**DOI:** 10.3390/ani9080540

**Published:** 2019-08-08

**Authors:** Michele Pazzola

**Affiliations:** Department of Veterinary Medicine, University of Sassari, via Vienna 2, 07100 Sassari, Italy; pazzola@uniss.it

**Keywords:** milk, coagulation, cheese yield, small ruminants

## Abstract

**Simple Summary:**

The suitability of milk for cheese production is usually predicted at dairy plants using mathematical formulas, which are based on milk protein and fat content. In addition, several methods, which use a small volume of milk to simulate the cheesemaking processes and stages, are currently available at the laboratory level. Those methods have been developed and improved for cow milk. In the present review, the author reports the available literature and current methods to analyze coagulation traits from sheep and goat milk.

**Abstract:**

Milk production from sheep and goat species is continuously growing worldwide, and its main use is for cheesemaking. Given that the final quality of cheese is linked to the traits of raw milk cheese yield at dairy plants, it is often calculated by using predictive formulas based on fat and protein content. Predictive formulas have been studied for bovine milk and are very effective but not appropriate for sheep and goat milk. Several methods, which simulate the actual coagulation processes, are available at the laboratories. This article reviews the available literature about rennet coagulation and cheese yield traits from sheep and goat milk and the methods used at the laboratory level. In general, if compared to cow milk, sheep and goat milk are characterized by shorter rennet coagulation times and a very limited amount of non-coagulating samples. Curd firmness of sheep milk is almost independent from the rennet coagulation time, and some coagulation traits can be predicted by infrared spectra. In addition, coagulation traits are characterized by appropriate values of heritability to be considered in selective breeding plans. With regard to goat milk, rennet coagulation time and cheese yield are strongly influenced by the breed effect.

## 1. Introduction

The total world production of milk from sheep and goats is constantly growing, and its main industrial use is for cheesemaking [1]. In some geographical areas, such as the Mediterranean Basin, although products are often differentiated in many local varieties, they are characterized by their high quality and are labelled with official designations, which identifies and enhances their specificity [2]. Quality of cheese is linked to characteristics of milk and to cheesemaking technologies. Cheese yield (CY%), which is calculated as the percentage of raw milk that is transformed into fresh curd, measures the efficiency of the cheesemaking process. The CY% is closely related to the concentration of fat and protein [3], and it is therefore predicted on the basis of fat and protein content. The current payment systems for sheep and goat milk in relation to quality must necessarily guarantee compliance with the minimum microbiological characteristics, but those also apply a bonus or a penalty to farmers according to the percentage of fat and protein [4]. Nevertheless, cheese yield and the final quality of the products cannot be solely attributable to the chemical composition of milk. In the past, several mathematical formulas have been developed for the prediction of those characters at the dairy industry level [5]. Although the use of those formulas represents a valid control tool for the dairy industry, laboratory methods can provide a useful and complete overview of the real coagulation process and the achievement of phenotypic data during many steps of the dairy chain.

Milk coagulation can be activated by many types of biochemical and physical processes. The most common coagulating agent at dairy plants is the rennet, which contains the chymosin enzyme. Chymosin starts the primary phase of milk coagulation, the proteolytic phase, breaking down the biochemical bond between the two amino acids phenylalanine and methionine, respectively, at positions 105 and 106 of the κ-casein protein chain. The event causes the destabilization of milk micellar structure. Later, during the secondary phase of coagulation, destabilized micelles precipitate and aggregate into a gel. The cutting of the gel into pieces, which is normally made during the cheesemaking processes, is the starting point of a phenomenon named syneresis, during which the network formed by the aggregated micelles facilitates whey expulsion and finally the formation of the fresh curd [6].

**Figure 1:**
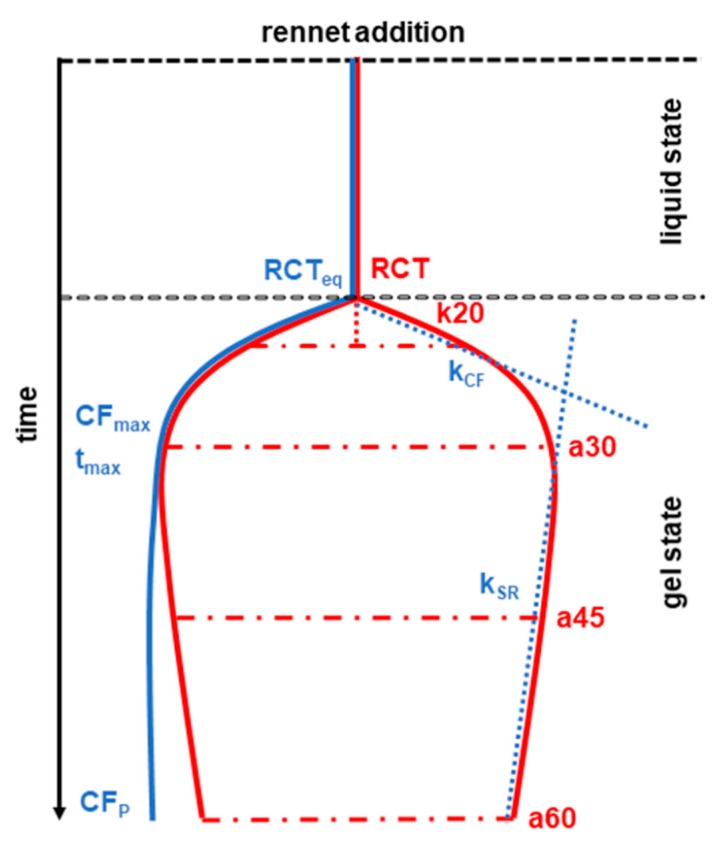
Graphic representation of milk coagulation traits based on the papers and illustrations by McMahon and Brown [8], Bittante [9], and Bittante et al. [10,11]. Traditional or single-point milk coagulation properties (MCPs) are reported in red: The rennet coagulation time (RCT) (measured in min) is i.e.,the interval between rennet addition and coagulation; k20, curd firming time (min), the interval between milk coagulation, and a width of 20 mm of the bell diagram; a30, a45, and a60, curd firmness (mm), the width of the bell diagram at 30, 45, and 60 min after rennet addition. Modeled curd firming over time (CF_t_) traits are in blue: Rennet coagulation time estimated by the CF_t_ equation (min) (RCT_eq_); the maximum potential curd firmness after an infinite time (mm) (CF_P_); curd-firming rate constant (% × min^−1^) (k_CF_), the higher the value, the faster the curd firming; syneresis rate constant (% × min^−1^) (k_SR_), the higher the value, the stronger the syneresis force; maximum curd firmness (mm) (CF_max_); time to attain CF_max_ (min) (t_max_).

Several techniques have been proposed and used to investigate the phases of milk coagulation, both at industrial and laboratory level. The most common devices are classified as mechanical, which exploit the drag force exerted by the clot; vibrational, based on the use of probes that vibrate in different ways as a response to milk coagulation; and optical, which record the changes of properties, color, and absorbance of milk during the coagulation phases [7]. Among the mechanical devices, the Formagraph instrument (Foss Electric A/S, Hillerød, Denmark) is very common at research and dairy plant laboratories. The Formagraph produces a graphic representation of the coagulation phases (Figure 1) based on the signals transmitted by steel pendula immersed in milk samples mixed with a rennet solution. The pendula record the changes of milk from the liquid to the gel state and the increase of curd firmness and syneresis [8]. The Formagraph method is based on the analysis of a small quantity of milk, 10 mL, and the achievement of the so-called traditional milk coagulation properties (MCPs, Figure 1): The rennet coagulation time (RCT), the curd firming time, k20, and the curd firmness, a30. 

The Formagraph method has some weaknesses [9,12]. It provides static phenotypes, which have been called “single-point” traits because those are recorded in a single moment and therefore do not provide the complete description of the coagulation process. In addition, traditional MCPs often show low repeatability, and many samples, for which no coagulation is recorded within the time period of 30 min, are labelled as “non-coagulating” (NC). Another negative aspect of the Formagraph, and in general of all the mechanical devices, is the loss of time, because 30 min are necessary for the recording of the MCPs from 10 milk samples.

Bittante [9] and Bittante et al. [10] proposed an extension of the Formagraph analysis up to 60 min and the elaboration of statistical models to examine the complete dataset extrapolated from the traditional Formagraph layout. Indeed, the instrument records the firmness of milk samples every 15 s for a total of 240 observations during 60 min, which are used to calculate the so-called traits of curd firmness over time (CF_t_) (Figure 1). The CF_t_ equation provides the estimated rennet coagulation time (RCT_eq_), the maximum potential curd firmness after an infinite time (CF_P_), the curd-firming rate constant (k_CF_), the syneresis rate constant (k_SR_), the maximum curd firmness (CF_max_), and the time to attain the maximum curd firmness (t_max_). Despite CF_t_ traits are not directly achievable from the Formagraph instrument, those can provide more dynamic data on the coagulation and syneresis processes and complete the information from the traditional MCP.

In order to improve the routinely measurement of MCP and CF_t_ traits, numerous studies investigated the possibility of an indirect prediction of coagulation traits through the calibration of the near and mid-infrared spectra [12,13,14]. Another recent development of the Formagraph method is represented by the laboratory method called the 9 mL milk cheesemaking assessment (9-MilCA). Through some modifications of the original Formagraph procedure and operations that simulate the cutting, pressing, and draining of the curd, the 9-MilCA allows the calculation of the CY% and the recoveries of milk nutrients in the curd [15].

Methods and papers described above have been developed and tested on cow milk, whereas the first studies dealing with small ruminant species date back to the late 80s [16]. The aim of the present review is to describe the state of the art and possible development of researches about coagulation properties of sheep and goat milk.

## 2. Sheep Milk

Sheep milk, when compared with other dairy species, is characterized by high protein and lipid content, at 5.7 and 6.9%, respectively [17]. World production of sheep milk is continuously growing and almost all is generally destined for cheesemaking, and direct consumption is rare [18]. Nonetheless, many aspects related to the study of coagulation characteristics are still unexplored. 

If we could measure the attractiveness of a topic in the scientific community, we would search, by means of the modern computerized engines, how much that topic has been studied in scientific documents. The research products available at an international search engine [19], after entering the key words “milk + coagulation + properties + species”, are 56 for sheep, in comparison with 256 for cattle. This is an expected finding and demonstrates a limited interest and economic value of sheep milk in the world of scientific research, but at the same time a positive perspective for potential future research relating to a topic that is still not fully investigated.

The first study to investigate prolongation of the Formagraph up to 45 min and the modeling of CF_t_ parameters was conducted to achieve characterization of milk from endangered sheep breeds from the Alps [20]. The comparison with cow milk has evidenced new information about the processes of ovine milk coagulation, which were not effectively available by the single-point MCPs, as with a higher value of k_CF_, which causes a much steeper increase in curd firming and a lower value of k_SR_, which causes a slower syneresis force.

An article based on the collection of a large number of individual samples presented the results concerning the MCPs of Sarda sheep milk recorded up to 60 min after rennet addition and the influence of individual and environmental effects [21]. The average RCT value, 8.6 min, demonstrated the significant difference with the bovine species. Indeed, with the use of the same method and protocol, the value for cow milk was between 10 and 20 min [22]. Another substantial difference was related to the very low percentage of non-coagulating ewe milk samples, 0.4%, validating the excellent curding ability of sheep milk. When non-coagulating samples are not properly computed, they can misrepresent the real meaning of the MCPs in a population. For example, in dairy specialized cattle breeds for the intensive production of milk, such as Holstein Friesian cattle, non-coagulating milk samples are very frequent and can bias the evaluation of genetic estimates of coagulation traits [10]. Some authors have suggested to overcome the negative influence of non-coagulating samples in cattle populations by selecting animals carrying the κ-casein B allele or other genetic markers associated with favorable coagulating traits [23]. The variability of sheep milk RCT is moderately influenced by the farming management and the different environmental conditions, while the stage of lactation, the level of production, and parity have more evident effects [21]. Moreover, k20 is faster, and a30, which is on average at 50 mm, is considerably higher than in cows. Indeed, the value of a30 recorded from Brown Swiss cattle, a breed characterized by an excellent cheesemaking ability, is at 30 mm [13,24]. Another characteristic of sheep milk is the almost complete independence of the value of a30 and RCT, with a phenotypic correlation between the two traits at −23%, whereas it is at −81% in cows [21,22]. Hence, high values of curd firmness are achievable when the change from the liquid to the gel state, measured by RCT, is delayed. In comparison with MCP data recorded from the Sarda breed, many differences attributable to the breed and the farming effects have been reported by other authors for local breeds from Spain [25], the Assaf [25], and Comisana [26]. 

As already evidenced for cattle [10], the application of non-linear statistical models to the dataset of sheep milk provided by the Formagraph showed that the single-point MCPs could be misleading to achieve a correct interpretation of the whole coagulation and syneresis processes. Indeed, CF_t_ traits of sheep milk were characterized by a high variability, with some samples presenting an extreme syneresis and others characterized by a nearly null syneresis [27]. 

Even if the main utilization of sheep milk is cheesemaking and the significant effects of genetic polymorphisms on MCP [28], there is no inclusion of coagulation traits in current genetic improvement programs of dairy sheep [25]. In fact, genetic plans of flocks’ books generally include traits related to milk yield, protein and fat contents, and, sometimes, the somatic cells count and udder morphology [29]. Recent researches suggested that, in the future, additional traits could be taken into account by novel selective programs. Bittante et al. [11] demonstrated that the heritability of coagulation traits, even if lower than those recorded in cattle, could be exploited with the best results for CF_t_ traits, namely for RCT_eq_, which showed a value of heritability at 0.317. Additionally, Manca et al. [30] reported some factors affecting coagulation traits with a moderately high value of heritability, as in the case of udder health scores. A further study investigating the effects of somatic cells, lactose, and pH and the significant effect on coagulation of sheep milk, confirmed the exploitable heritability values of those markers and the potentiality to be computed in genetic schemes, together with MCPs [31]. 

Some restrictions for the suitable achievement of data and the useful insertion of coagulation traits in sheep genetic schemes are represented by the protocol of mechanical devices, since it takes at least 30 min for processing each individual sample. Indeed, the economic ratio between the price of raw milk produced at the farms and the charge of MCP and CF_t_ analysis is still unfavorable [11]. As described in the Introduction paragraph, the analytical technique that can allow a significant reduction of costs is represented by the infrared spectroscopy [12,13,14], which is the method commonly used at milk laboratories for the prediction of several traits such as fat, protein, and pH. The investigation and calibration of the spectra, generated by infrared spectroscopy equipment, is therefore exploitable to indirectly measure coagulation traits at the individual [32] and bulk milk level [26]. For individual ovine milk samples, the correlation between the predicted value by means of the spectrum and the value measured by the Formagraph showed reliable values; the highest value for RCT at 0.88 and repeatability measures often above 95% [32].

The recent use of the 9-MilCA procedure [15] on sheep milk permitted to investigate the cheese yield, cheesemaking efficiency, nutrient recovery, and the relationship with milk composition and coagulation traits. Vacca et al. [33] highlighted that the mean value of sheep milk CY%, i.e., the ratio between raw milk submitted to coagulation and fresh curd, was at 25.8%—the CY% was highly influenced by fat and protein, as expected, but also by lactose—and that the curd firming instant rate, k_CF_, was the most informative trait to investigate the cheesemaking efficiency. At the laboratory level, the use of the 9-MilCA was suitable for mimicking the real processes used at dairy plants and avoided the bias linked to methods based on curd centrifugation to allow whey separation and the consequent overestimation of cheese yield [34,35].

## 3. Goat Milk

Goat farming is in continuous expansion, both in developing countries and in those with a higher level of industrial production [2]. Goat milk is used in a versatile way [36]. Marketing for direct consumption as fluid milk, which is well accepted because of the low allergenicity, and dairy processing are the prevalent industrial utilizations [17]. 

At the present time, the scientific literature regarding coagulation traits of caprine milk is represented by 69 available documents [19]. These articles are mainly focused on the analysis of a single breed, on the association with the polymorphisms of the genetic cluster of caseins, and often with a limited number of animals. 

Recent articles by Vacca et al. [37] and Stocco et al. [38] investigated coagulation properties of goat milk and the influence of milk composition, environmental, and individual effects. In those studies, the use of a common protocol allowed for a comparison of the properties of goat milk with other dairy species. The average value of RCT recorded for the goat, 13.2 min, was similar to the Mediterranean buffalo and intermediate to sheep and cattle. The mean curd firmness a30 was 36 mm, and the prolongation of the analysis up to 60 min revealed a marked syneresis starting at 45 min after rennet addition. Similarly, to sheep, the authors reported a limited number of non-coagulating samples, at 1%. The variability of the MCPs was significantly influenced by the management: 37% of the total variability was attributable to the farm effect for RCT and 69% for curd firmness a60. As regards the effect of milk nutrients, the higher the fat content, the better the coagulation properties, the higher the protein content the more delayed the RCT, and the higher the casein content the higher the curd firmness.

The modeling of CF_t_ traits demonstrated that the high incidence of the farm effect was mainly linked to the different farming techniques, e.g., extensive vs. intensive ones [39]. Other phenotypic effects that significantly influenced coagulation traits were parity and, above all, the breed. In fact, although daily milk yield was obviously higher in specialized breeds, such as the Saanen and the Murciano–Granadina, the curd firmness was higher in native breeds, such as the Sarda goat, with a mean value of a30 at 40 mm. That last result suggested a higher dairy efficiency per liter of milk for native breeds. A statistical model used to quantify the direct effect of the breed has showed that the differences are due to the preponderant direct effect of the breed for some traits, such as for RCT, and to the indirect effect of the milk composition for others, such as for the curd firmness [37].

The use of the 9-MilCA method [15] provided novel information about the prediction of cheesemaking traits from goat milk. In general, protein recovery in the curd was higher than in other species, with an average value at 81%. The CY% showed marked differences between specialized and autochthonous breeds, with values ​​ranging from 13.6 for the Saanen and 17.8% for the Sarda, and high values of fat and protein contents in milk improved the CY% and the recovery of all milk nutrients in the curd [40,41]. 

## 4. Conclusions

The characteristics highlighted for sheep and goat milk indicate that information from other species cannot be fully applied to small ruminants. The results are suitable to support the enhancement of production potential at farms and dairy plants. Nevertheless, further studies should investigate many aspects of this topic, which are still to be clarified:

1. The differences among small ruminant breeds and farming techniques are still marked. Indeed, differently than cattle dairy sector, in which intensive farming and Holstein–Friesian cows are prevailing worldwide, sheep and goat milk productions are made from numerous breeds, both specialized and local ones, in large intensive or small extensive farms. In this scenario, a general application of results achieved from a specific breed and farming method could be very limited.
2. The influence of individual samples on coagulation ability of bulk milk destined to dairy plants.
3. The actual utilization of faster and cheaper methods to achieve coagulation traits and the introduction of these traits in genetic schemes and milk payment systems at the cheese industry level [42].
